# Current Trends in Non-Invasive Imaging of Interactions in the Liver Tumor Microenvironment Mediated by Tumor Metabolism

**DOI:** 10.3390/cancers13153645

**Published:** 2021-07-21

**Authors:** Isabel Theresa Schobert, Lynn Jeanette Savic

**Affiliations:** 1 Department of Radiology, Charité-Universitätsmedizin Berlin, Corporate Member of Freie Universität Berlin, Humboldt-Universität zu Berlin, and Berlin Institute of Health, 10117 Berlin, Germany; isabel.schobert@charite.de; 2 Berlin Institute of Health, 10178 Berlin, Germany

**Keywords:** tumor metabolism, tumor microenvironment, immune system, positron emission tomography, magnetic resonance spectroscopy, optical imaging, targeted therapies

## Abstract

**Simple Summary:**

Deregulated tumor metabolism is known to shape the tumor microenvironment and directly affect the local immune response, promoting tumor growth, metastasis, and resistance to treatment. However, the metabolic profile or tumor cells, and therefore, the composition of their microenvironment, are highly variable among patients and even within the same tumor, resulting in heterogeneous response rates to oncologic therapies, making patient selection a key issue. This review article focuses on non-invasive imaging techniques that aim to visualize the crosstalk between tumor cells and their microenvironment in liver cancer mediated by tumor metabolism. In addition to improved tumor detection, such imaging tools may be able to provide a more accurate characterization of the individual tumor and ultimately improve understanding, as well as guide personalized treatment regimens for patients with liver cancer.

**Abstract:**

With the increasing understanding of resistance mechanisms mediated by the metabolic reprogramming in cancer cells, there is a growing clinical interest in imaging technologies that allow for the non-invasive characterization of tumor metabolism and the interactions of cancer cells with the tumor microenvironment (TME) mediated through tumor metabolism. Specifically, tumor glycolysis and subsequent tissue acidosis in the realms of the Warburg effect may promote an immunosuppressive TME, causing a substantial barrier to the clinical efficacy of numerous immuno-oncologic treatments. Thus, imaging the varying individual compositions of the TME may provide a more accurate characterization of the individual tumor. This approach can help to identify the most suitable therapy for each individual patient and design new targeted treatment strategies that disable resistance mechanisms in liver cancer. This review article focuses on non-invasive positron-emission tomography (PET)- and MR-based imaging techniques that aim to visualize the crosstalk between tumor cells and their microenvironment in liver cancer mediated by tumor metabolism.

## 1. Introduction

Hepatocellular carcinoma (HCC) is the most common primary liver malignancy, and primary liver cancer is the third most common cause of cancer-related death worldwide [1]. Incidence rates continue to increase, and the majority of HCC patients present at intermediate to advanced disease stages [2]. HCC mostly develops in cirrhotic, chronically inflamed liver parenchyma, with the main risk factors being chronic hepatitis B and C infection or alcoholic or non-alcoholic fatty liver disease (NAFLD) [2]. The outcome of patients largely depends on early detection and diagnosis, as well as on determining the most appropriate individual treatment and monitoring the response.

In routine clinical settings, anatomical imaging, such as contrast-enhanced computed tomography (CT) or magnetic resonance imaging (MRI), are used as standards of care for the detection and diagnosis, staging, and response assessments of HCC after treatment [3]. The characteristic radiological enhancement pattern of HCC is the pronounced contrast-uptake in the hepatic arterial phase and the subsequent wash-out in the portal venous and delayed phases, which is due to the pathophysiologic hypervascularization and the predominance of arterial over portal venous blood supply [4].

In terms of imaging quality, multiphasic MRI provides better soft tissue contrast than CT, allowing for a more detailed evaluation of nodules and background liver tissue characteristics [3]. The vast majority of MRI contrast agents are based on gadolinium, which shortens the T1 and T2 relaxation times of tissues in which it accumulates. In contrast to extracellular gadolinium-based contrast agents, MRI with hepatocyte-specific contrast agents is generally more sensitive in detection, but at the cost of a decreased specificity when diagnosing small HCC (<2 cm) [3]. Moreover, MRI is currently acquired clinically solely for qualitative diagnosis, but is not optimized for quantification, limiting its reproducibility and accuracy [3,4]. 

Alterations in metabolic tumor characteristics may become detectable earlier after treatment than changes in hypervascularization. Therefore, more comprehensive approaches to characterize and monitor the tumors metabolism are urgently needed [5]. 

In this context, the individual physiological and biological variations of the tumor microenvironment (TME) are proposed to play an important role over the dynamic course of carcinogenesis and reflect the aggressiveness of cancer cells by their changing composition [6,7]. Overall, these microenvironmental characteristics are associated with chemoresistance to multiple commonly used, systemically applicable, anticancer agents, as well as poor clinical outcomes [6,7].

Specifically, key functions of tumor metabolism resulting in the acidification of the TME significantly contribute to the building and preservation of a pro-tumorigenic niche [6]. The most frequently studied metabolic characteristic of cancer was first described by Otto Warburg as aerobic glycolysis, and comprises an increased glucose uptake and glycolytic metabolization even in the presence of sufficient oxygen instead of oxidative phosphorylation [8]. High glucose uptake and increased glycolytic activity have also been reported for HCC [9]. The aerobic glycolysis is faster but less energy efficient; thus, the cancer cell is required to perform glycolysis at a higher rate. The so-called “Warburg effect” leads to an accumulation of glycolysis by-products, such as lactate, with concomitant acidification of the TME, which, in turn, impairs the anti-tumoral immune response and promotes neo-angiogenesis and metastasis, all of which support tumor growth [10,11,12,13].

In addition to the dysregulated glucose metabolism, numerous other deranged metabolic pathways have been discovered in HCC as well as in other tumors, including the metabolism of amino acids and lipids [6,14]. However, the metabolic profile of tumor cells and the composition of the TME are highly variable among patients and even within the same tumor; therefore, they are unpredictable, resulting in heterogeneous response rates to oncologic therapies, making patient selection a key issue [12,15,16].

Thus, there is an unmet clinical need for novel imaging biomarkers that visualize the tumor and its microenvironmental characteristics, and thereby better reflect the true biochemical and pathological disease profile. In this regard, metabolic pathways have gained increased interest as targets for imaging and therapies [7]. This review article focuses on non-invasive positron emission tomography (PET)- and MR-based imaging techniques that aim to visualize the crosstalk between tumor cells and their microenvironment in liver cancer mediated by tumor metabolism (Table 1).

## 2. I Imaging of Metabolic Substrates and Flux

### 2.1. Glycolysis

#### 2.1.1. Glucose

**Figure 1:**
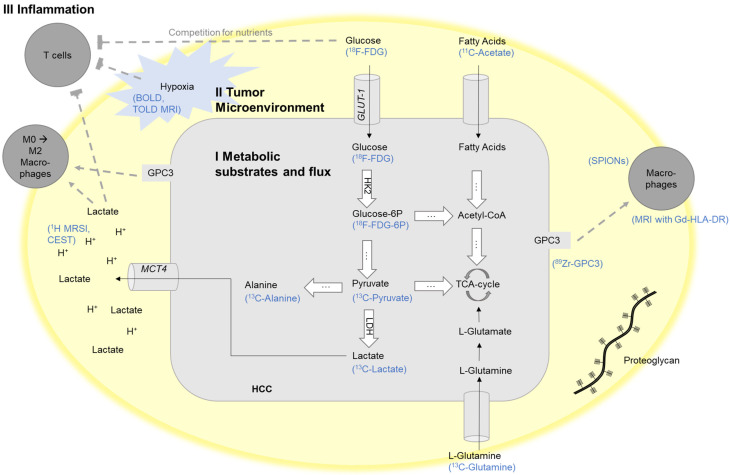
Schematic illustration of exemplary targets for the imaging of the crosstalk between the tumor cells and their tumor microenvironment mediated through tumor metabolism. BOLD, blood-oxygen-level-dependent MRI; CEST, chemical exchange saturation transfer; FDG, fluorodeoxyglucose; Gd-HLA-DR, gadolinium-conjugated antibodies against HLA-DR; HK-2, hexokinase-2; GAG, glycosaminoglycan; Glucose-6P, Glucose-6-phosphate; GLUT1, glucose transporter 1; GPC3, glypican-3; HCC, hepatocellular carcinoma; LDH, lactate dehydrogenase; MCT4, monocarboxylate transporter 4; MRI, magnetic resonance imaging; MRSI, magnetic resonance spectroscopic imaging; SPIONs, superparamagnetic iron oxide nanoparticles; TCA-cycle, tricarboxylic acid cycle; TOLD, tissue oxygenation level-dependent MRI. Adapted and modified from [37].

The most widely applied metabolic imaging in clinical routine is ^18^F-fluorodeoxyglucose positron emission computed tomography (^18^F-FDG PET), visualizing the uptake of the glucose analogue as an indicator of (upregulated) glycolysis in tumor cells (Figure 1). In clinical practice, PET is performed as hybrid imaging, combined with either CT or MRI, in order to be able to fuse the functional image information to the anatomical image. The ^18^F-FDG-signal can be quantified as a standard uptake value (SUV). However, besides the glucose uptake and tumor perfusion, there are also some HCC-specific factors resulting in the varying sensitivity of SUV [35,36].

HCCs mostly display a hyperglycolytic phenotype, and in order to maintain high rates of glycolysis, the glucose transporters GLUT-1 and -2 are upregulated, allowing for an increased uptake of glucose. Increased GLUT-1 expression has been correlated with worse prognosis in patients with HCC [9]. Inside the cell, glycolysis starts with the phosphorylation of glucose to glucose-6-phosphate with the enzyme hexokinase (HK). The most common isoform in HCC is HK2, which is also upregulated in hypoxia, for example, a common phenomenon in growing tumors [38]. In an HCC mouse model, HK2 knockdown inhibited glycolysis and thus glucose flux to pyruvate and lactate, and simultaneously increased oxidative phosphorylation, which could be diminished by metformin, contributing to further cell death and the inhibition of tumor growth [39]. Additionally, HK2 silencing also synergized with sorafenib, a multi-tyrosine kinase and angiogenesis inhibitor, to inhibit tumor growth [39].

In contrast to the overexpression of HK-2 in HCC, normal liver parenchyma expresses HK-4 most abundantly [6]. FDG follows the first steps of the metabolic pathway of glucose and is trapped inside the cell by phosphorylation; therefore, the retention of FDG inside the cell and the increased SUVs require the high expression of HK and low expression of glucose-6-phosphatase (G6P). As opposed to HCC, liver parenchyma has high levels of G6P and low HK. However, depending on the differentiation of HCC, expression levels of HK and G6P differ, leading to varying sensitivity of FDG-PET in HCC between 50% and 65% [40,41]. In highly differentiated, low-grade HCC, the enzymatic activity resembles that of normal hepatocytes, HK levels are usually low and G6P are levels high; thus, FDG retention is low and the sensitivity of ^18^F-FDG-PET/CT for the detection of HCC is therefore decreased. Additionally, signal quantification is limited by a decreased signal-to-noise ratio due to the FDG-uptake of liver parenchyma and the resulting background signal. These obstacles have so far prevented FDG-PET applications from being included in the routine management of HCC, although it may offer an opportunity to investigate HCC differentiation and distant metastases, which are most likely of poorly differentiated tumors [17].

#### 2.1.2. Pyruvate, Lactate, and Alanine

Pyruvate kinase mediates the last step of glycolysis, generating pyruvate and adenosine diphosphate. Pyruvate, in turn, can be further converted to lactate (by lactic dehydrogenase, LDH) or alanine (by alanine transaminase, ALT), or it can enter the tricarboxylic acid cycle (Figure 1).

In human HCC, LDH activity is up-regulated and serum LDH levels have shown a prognostic value in predicting overall and progression-free survival in HCC [42,43]. Another study reported that high serum levels of LDH correlated with worse median progression-free survival (PFS) in patients with HCC treated with sorafenib [44]. Thus, the non-invasive monitoring of glycolysis, and particularly the alternative conversion of pyruvate into lactate, could serve as a prognostic biomarker or tool for the monitoring of treatment responses.

Imaging of pyruvate and its predominant downstream metabolites can be used to differentiate tissues. In contrast to PET visualizing only tracer uptake, dynamic nuclear polarization magnetic resonance spectroscopic imaging (DNP-MRSI) enables the real-time visualization of the enzymatic conversion of the parent substrate to its downstream metabolites. DNP-MRSI utilizes hyperpolarized ^13^C-labeled substrates, which increases the sensitivity of conventional MRS. Using sequential scanning, this technique thus enables the quantitative assessment of metabolic flux (Figure 1) [45].

However, pyruvate DNP-MRSI requires higher doses of the infused hyperpolarized substrates, potentially interfering with metabolic flux and thus making the interpretation of the imaging findings more complicated as compared to PET imaging, which requires lower amounts of radiotracer.

Aerobic glycolysis and tissue acidosis are common phenomena in the TME; however, glycolytic rates and consecutive lactate accumulation can increase further in cases of tumoral hypoxia, which occurs in a majority of growing malignancies whose size exceeds the oxygen provided by tumor-supplying vessels. Additionally, tissue hypoxia may be severely exacerbated following embolotherapies, including transarterial (chemo)embolization (TAE, TACE), which constitutes a guideline-approved treatment for intermediate-stage HCC [46,47]. TAE is considered to be an intra-arterial embolization therapy that causes tissue infarction and ischemic insults within the treated lesions. One preclinical study showed that after TAE-induced ischemia, latent HCC cells demonstrated reduced metabolism and developed a dependence on glycolytic flux to lactate, which could be imaged by DNP-MRSI of 1-^13^C-pyruvate and its downstream metabolites, 1-^13^C-lactate and 1-^13^C-alanine, which, in turn, predict the histological viability of treatment-refractory HCC cells after TAE [20].

In a recent study using a rat model of HCC, DNP-MRSI after 1-^13^C-pyruvate injection revealed a higher peak lactate-to-alanine signal ratio in HCC compared to non-tumorous tissue. Dynamic DNP-MRS revealed higher pyruvate-to-lactate conversion rates and lactate signals in tumors derived from more hyperglycolytic cells, validated by ex vivo measurements of higher lactate dehydrogenase levels [19]. Investigations in an orthotopic rat model of HCC demonstrated that the conversion of 1-^13^C-pyruvate to 1-^13^C-alanine was significantly higher than the conversion of 1-^13^C-pyruvate to 1-^13^C-lactate [48].

A study from Düwel et al. investigated a more comprehensive multiparametric DNP-MRSI approach in HCC-bearing rats before and after TAE. Measurements included hyperpolarized urea, revealing information about tumor perfusion (measured as tumor-to-muscle and tumor-to-liver ratios of urea), pyruvate and its metabolic conversion, as well as fumarate conversion to malate, providing insight into the levels of necrosis [49]. However, due to the cellular export and re-circulation of lactate, systemic lactate levels should be taken into account when modelling local conversion rates [50].

### 2.2. Lipid Metabolism

The liver metabolizes lipids and lipoproteins and is of key importance in the synthesis, storage, and degradation of lipids. Additionally, cancer metabolism displays dysregulated de novo lipogenesis, a pathway that controls the biosynthesis of fatty acids [51]. In cases of glucose depletion, fatty acid oxidation can be used to provide additional energy for cell survival and proliferation [52]. Dysbalanced lipid metabolism, as in patients with obesity, diabetes and hepatic steatosis, is associated with an increased risk of developing HCC. Type 2 diabetes mellitus increases the risk of HCC development; thus, metformin treatment substantially decreases the risk of HCC development and progression in a dose-dependent manner [53]. Consequently, there is currently a phase III clinical trial investigating metformin treatment in patients with HCC (NCT03184493). Additionally, statins (β-Hydroxy β-methylglutaryl-CoA (HMG-CoA) reductase inhibitors), which are used to lower cholesterol levels, can attenuate the risk of HCC development in patients with or without diabetes and HCC [54].

#### 2.2.1. Choline

Only very limited data regarding the imaging of lipid metabolism in HCC exist. The most frequently investigated technique is PET imaging using choline as a radiotracer. Choline is needed for cell membrane phospholipids. Increased proliferation rates in cancer require the increased metabolism of cell membrane components, leading to enhanced choline uptake and utilization [55,56]. A meta-analysis demonstrated an average detection rate of ^11^C-choline PET of 84% for HCC across five studies [57]. However, particularly for a subset of HCCs, which were moderately differentiated, these showed relatively high choline uptakes and thus a better detection rate with ^11^C-choline PET (75% detection sensitivity) as compared to poorly differentiated HCC with markedly lower choline uptake (25% detection sensitivity) [21]. This uptake behavior is inverse to the FDG-uptake; therefore, another study prospectively investigated the benefit of combining ^11^C-choline with ^18^F-FDG PET and showed a markedly increased sensitivity for detecting HCC of 93% (vs. choline PET alone 75% and FDG PET alone 36%) [22].

#### 2.2.2. Acetate

Acetate can be used by tumors as a substrate in de novo fatty acid synthesis. The method of ^11^C-acetate PET imaging showed an encouraging sensitivity of 75% for detecting HCC, but decreased to 32% in HCCs smaller than 2 cm [24]. Similar to ^11^C-choline, well-differentiated HCC tumors were detected by ^11^C-acetate, demonstrating a benefit of the complementary use of acetate together with FDG PET imaging as well. Additionally, non-HCC malignant liver lesions did not show increased ^11^C-acetate uptake and of the benign liver lesions that have been investigated only FNH showed mildly increased ^11^C-acetate uptake, suggesting a high specificity [58]. Dual tracer (^11^C-acetate and ^18^F-FDG) PET/CT was superior to contrast-enhanced CT in detecting HCC in cirrhotic liver parenchyma [23].

#### 2.2.3. Amino Acids

When compared to hepatocytes, HCC cells exhibit an increased uptake and metabolism of glutamine, which has been shown to correlate with tumor progression [59]. Thus, glutamine may serve as another interesting target for metabolic cancer imaging. Among other preclinical approaches, the conversion of glutamine to glutamate was demonstrated with hyperpolarized 5-^13^C(1) glutamine MRS measurements in human HCC cells and in a rat model of HCC [25,60].

### 2.3. II Tumor Microenvironment

#### 2.3.1. Extracellular pH

The mostly hyperglycolytic tumor phenotype of HCC leads to an accumulation of lactate and protons, which need to be excreted in the extracellular milieu in order to maintain the intracellular pH and dependent cellular functions. In cancer cells, intracellular pH (pH_i_) is increased as compared to normal cells (7.2–7.5), whereas extracellular pH (pH_e_) is usually more acidic (6.5–6.9) [61]. Extracellular tumor acidosis is associated with more aggressive tumor growth and invasion, neoangiogenesis and metastasis, and chemoresistance to, e.g., doxorubicin [61,62,63,64,65,66]. The increased expression of carbonic anhydrases, monocarboxylate transporter 1 and 4, and Na^+^-H^+^ exchanger 1, facilitate the efflux of protons and lactate into the extracellular milieu [67,68,69,70]. The subsequent acidic pH_e_ in the TME can serve as a biomarker for oncologic imaging to visualize the result of enhanced glycolysis as well as a therapeutic target in order to mitigate the resistance mechanisms to chemotherapy or radiation (Figure 1).

Currently, various pH probes exist that generally use the physical properties of acidic protons for MRS and MRI. Chemical exchange saturation transfer (CEST) saturates exchangeable protons which are transferred to bulk water signals and can be used to measure pH_e_. Ioversol CEST MRI has been successfully used for pH_e_ mapping of the liver cancer microenvironment in a rat hepatoma model [26]. Radiofrequency (RF) power-based ratiometric CEST (dual-power CEST) MRI using ioversol has been shown to be able to differentiate human HCC from benign hemangioma based on measurements of the pH_e_ on a 3T MR scanner [27]. In a model of mammary cancer, CEST-fast imaging with a steady-state free precession technique (CEST-FISP) MRI method was applied to detect the CEST of two amide protons of iopromide, a clinically used CT contrast agent. This adapted CEST method was then used as a non-invasive and relatively fast imaging technique to detect the change in tumor environmental pH_e_ after bicarbonate treatment (Figure 1) [71].

**Figure 2:**
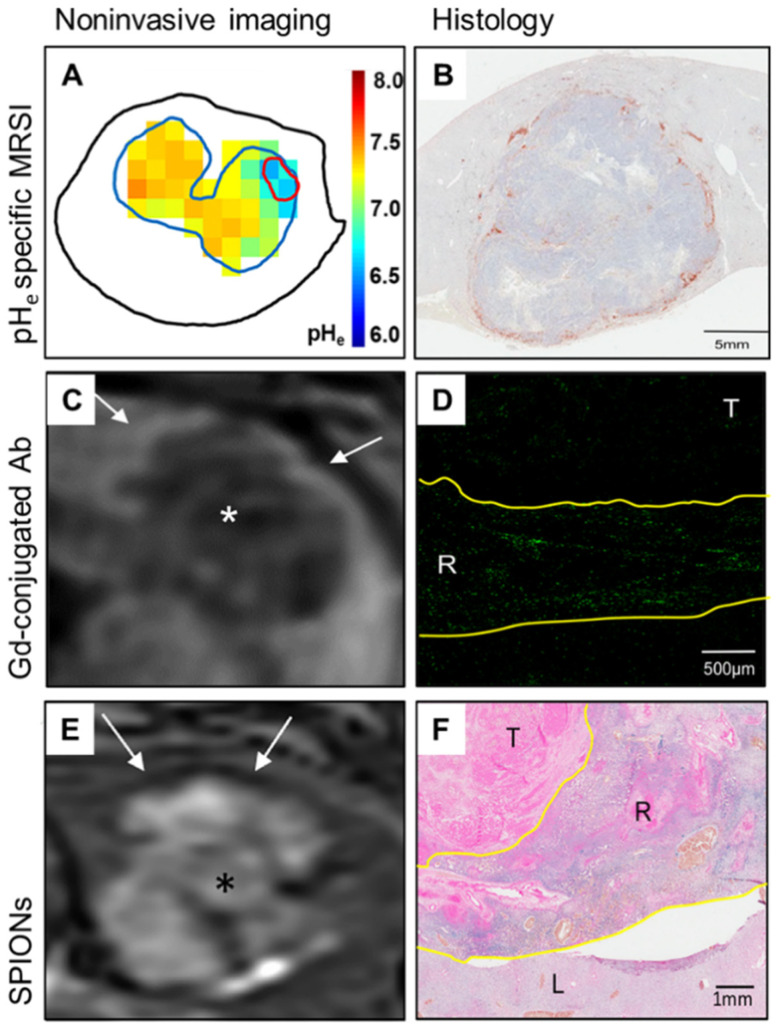
Imaging of the immuno-metabolic crosstalk in a translational rabbit liver tumor model. (**A**) Non-invasive quantitative mapping of the extracellular pH (pH_e_) using pH_e_-specific magnetic resonance spectroscopy imaging (MRSI) reveals significantly lower tumor pH_e_ as compared to the liver parenchyma. (**B**) Immunohistochemistry staining of HLA-DR on antigen-presenting cells demonstrates peritumoral immune cell clusters and lacking intratumoral infiltration. (**C**) T1-weighted MR imaging (MRI) after the injection of gadolinium (Gd)-conjugated HLA-DR antibodies (Ab) shows corresponding peritumoral rim enhancement in vivo. (**D**) Ex vivo imaging mass cytometry confirms localization of the labeled immune cells in the peritumoral rim in green, (**E**) T2-weighted MRI with superparamagnetic iron oxide nanoparticles (SPIONs) demonstrates hypointense signal indicative of macrophage infiltration in the peritumoral rim. (**F**) Ex vivo Prussian blue staining confirms corresponding iron deposition in the peritumoral rim. T, tumor; R, tumoral rim; L, liver parenchyma; white arrows, signal alterations at the tumoral rim; *, tumor. Adapted and modified from [34].

Another MR-based approach is based on the MRSI of the pH-dependent chemical shifts of ^1^H or ^31^P. Biosensor imaging of redundant deviation in shifts (BIRDS) is a relatively fast voxel-based MRSI technique measuring ^1^H signals, which detects paramagnetically shifted non-exchangeable protons from lanthanide Ln^3+^ complexes that are independent of contrast agent concentration in the investigated tissue. This technique has been validated in translational organotypic 3D culture models of liver cancer in vitro as well as in vivo using the orthotopic VX2 rabbit liver tumor model. Specifically, untreated VX2 tumors showed significantly lower pH_e_ than the surrounding liver parenchyma. Within two weeks after TACE, tumor pH_e_ gradually increased towards normal liver parenchyma pH_e_, suggesting that tumor pH_e_ can serve as a functional biomarker for tumor responses to non-surgical therapies of liver cancer (Figure 1 and Figure 2) [5,28].

In addition to pH_e_ mapping, there are theranostic approaches based on selective targeting of the acidic tumor pH_e_, such as the acidic pH-triggered drug-release of sorafenib and superparamagnetic iron oxide nanocomposites. These nanocomposites showed good iron-mediated MRI contrast, while simultaneously significantly inhibiting tumor growth in a rat model of HCC [29]. In another study, pH-responsive lactosylated nanoparticles containing sorafenib and curcumin were injected i.v. in an HCC xenograft model and showed a reduction in tumor growth [72].

More recently, a new PET-based method for selective pH imaging was introduced. ^18^F-FDG amine, an acid labile prodrug ^18^F-FDG derivative, is selectively degraded to the parent compound upon exposure to acidic pH and can be taken up by adjacent cells, as with conventional ^18^F-FDG, and subsequently be imaged by PET [30]. This approach aims to reduce the problem of background signals in FDG-PET, potentially making it more applicable, e.g., in the brain, kidneys or the liver.

#### 2.3.2. Hypoxia

Tumor hypoxia drives the hyperglycolytic phenotype of cancer cells and the consecutive accumulation of lactate and protons, exacerbating the low pH_e_ [73]. Additionally, hypoxia results in the upregulation of various growth factors, e.g., hypoxia-inducible factor (HIF)-1α, which promotes angiogenesis, endothelial-to-mesenchymal transition, and thus, metastasis in cancer [74,75,76,77]. It further propagates the expression of immune checkpoints, facilitates regulatory T cell activation, and promotes macrophage polarization towards an anti-inflammatory, pro-tumorigenic M2 phenotype, all of which contribute to immune evasion of the tumor, resistance to radiation and systemic therapies, and ultimately, a poor prognosis (Figure 1) [76,78].

Imaging hypoxia in the TME may allow for the identification, spatial mapping and quantification of tumor hypoxia prior to treatment as well as the monitoring of treatment-induced alterations, e.g., following embolotherapies. However, no hypoxia imaging methods are currently being used in clinical practice.

Several PET-based imaging techniques exist, but have been mostly studied in tumor entities other than HCC, i.e., head and neck cancer. Head and neck cancers are often treated with radiotherapeutic approaches; therefore, the non-invasive localization of foci of tumor hypoxia with hypoxia-specific PET tracers, such as ^18^F-fluoromisonidazole (^18^F-FMISO), could be useful to guide the targeted therapy of radioresistant hypoxic tumors [79].

In addition to PET-based techniques, several MRI-based approaches for the imaging of hypoxia have been studied in HCC. Those techniques either measure the changes in longitudinal relaxation (R(1)), the tumor oxygenation level-dependent (TOLD) MRI, the changes in effective transverse relaxation (R(2)*), or the blood-oxygen-level-dependent (BOLD) MRI induced by inhalation of either oxygen or carbogen [80]. A study investigating these techniques in 34 human HCCs found a decrease in R2* and an increase in R1 after the inhalation of oxygen as expected with increased tissue and blood oxygenation [81]. In a rabbit VX2 liver tumor model, BOLD-fMRI detected a decrease in T2* after TACE, consistent with the therapeutic tumor hypoxia induced after embolization [82]. Another study investigated the BOLD response following oxygen inhalation in cirrhotic liver parenchyma, which has abnormal vascular autoregulatory mechanisms and therefore shows an elevated BOLD response [31].

#### 2.3.3. Extracellular Matrix

In addition to energy generation, glucose plays a pivotal role in shaping the extracellular matrix (ECM), which provides structural and biochemical support to tumor cells as well as the surrounding cells of the TME [83,84]. In addition to collagen and elastin, proteoglycans, a subclass of glycoproteins, are a major component of the ECM. They contain at least one glycosaminoglycan chain which is attached to the core. They are located in the ECM, on the cell surface, and in the cytoplasm.

Glypican-3 (GPC3), a cell surface-linked heparan sulfate proteoglycan, is physiologically present only in the fetal liver but not in adult liver parenchyma [85]. However, GPC3 is upregulated in a variety of tumor entities including HCC, and its expression is reportedly associated with poor prognosis. Its main functions in cancer progression are the GPC3-induced stimulation of Wnt signaling for tumor progression, the interaction of GPC3 with various growth factors, the stimulation of epithelial–mesenchymal transition (EMT), and the recruitment and polarization of macrophages towards a tumor-promoting M2-phenotype (Figure 1) [86,87,88,89,90,91,92,93,94,95].

Given its high tumor-to-liver ratio, GPC3 is supposedly suitable for targeted imaging approaches in HCC. For PET imaging, GPC3-labeled ^89^Zr was able to detect HCC in liver parenchyma in an orthotopic xenograft mouse model. To reduce the long circulation time and therefore the background signal of the monoclonal antibody, an αGPC3-F(ab′)2 fragment conjugated to ^89^Zr was developed and yielded significantly accelerated blood clearance and increased signal-to-noise ratios [32].

Regarding GPC3-specific MRI probes, an anti-GPC3-ultrasuperparamagnetic iron oxide probe for early HCC detection was developed and exhibited specific uptake behavior of HepG2 cells and decreased signal intensity in T2-weighted images in vitro [96]. In a clinical study, a multifunctional nanoparticle specifically binding GPC3 was used in pretreatment MRI for HCC detection, and additionally visualized HCC during operation through near-infrared fluorescence [97].

Although broad clinical applications remain warranted, GPC3-targeted imaging may have the potential to complement the staging of HCC, the identification of the tumor margin during surgery, as well as the monitoring after treatment.

### 2.4. III Inflammation

#### 2.4.1. Immune Evasion

Chronic liver diseases not only display altered metabolism, but also generate a chronic proinflammatory milieu, allowing for hepatic tumor formation and progression [98]. In HCCs, the TME consists of stromal cells, hepatic stellate cells, and endothelial and local immune cells. The crosstalk between tumor cells and their surrounding microenvironment is required for sustaining HCC development and progression by promoting (neo-)angiogenesis and EMT [98].

The TME constitutes a harsh environment for infiltrating immune cells due to the competition for nutrients and metabolic by-products of accelerated tumor growth requiring the immune cells to adapt. Cancer cells mostly rely on aerobic glycolysis; proinflammatory M1 macrophages and activated T cells also shift their metabolism towards an activated state with increased nutrient uptake, glutaminolysis, and aerobic glycolysis (over oxidative phosphorylation), which is needed to fuel their effector functions [99,100]. M1 macrophages rely on aerobic glycolysis as well [101]. This results in competition for substrates between cancer cells and immune cells, where cancer cells usually outpace the local immune cells in the TME. Moreover, the metabolic tumor by-products, such as lactate, and tumor hypoxia, further contribute to the immunosuppressive, pro-tumorigenic niche, in which immune cells need to adapt in order to survive [102,103].

Active inflammatory stimuli promote the polarization of macrophages towards an M1-like phenotype, which, in turn, produces inflammatory cytokines. In contrast, anti-inflammatory stimuli induce the polarization towards an M2-like phenotype with immunosuppressive functions. During chronic inflammation, e.g., in viral hepatitis, the immunosuppressive M2-like macrophage phenotype becomes more prevalent [104,105]. The interplay between M2-like tumor-associated macrophages (TAM) and HCC is complex. Tumor-derived lactate enhances M2 polarization and the expression of vascular endothelial growth factor (VEGF) of TAMs [106]. Moreover, the accumulation of lactate and acidification of the TME reduces the interferon-gamma expression of cytotoxic T cells and natural killer cells [73].

Additionally, it has been shown that M2-like TAMs promote EMT and chemoresistance in HCC and are associated with poor overall survival in HCC [107,108]. This highlights the importance of targeting the immune microenvironment with tailored imaging techniques and therapeutic approaches.

One study with six patients, one of them with HCC, investigated a non-invasive PET/CT-based technique with radiolabeled minibodies against CD8+ T cells, and found this approach to be safe and suitable for early imaging. The biodistribution was highest in spleen and bone marrow, but PET/CT showed accumulations in tumors as well [33].

Moreover, quantitative T2-weighted magnetic resonance imaging, using superparamagnetic iron oxide nanoparticles (SPIONs), has been used for the non-invasive imaging of TAMs in animal models and in human trials [34]. The SPIONs are slowly phagocyted by macrophages and decrease T2 MR relaxation times in inflamed tissue or tumors several hours after the injection of SPIONs. Imaging of TAMs using SPION is a promising method for the assessment of the inflammatory response to the tumor and tumoral immune cell infiltration, and may be used for the evaluation of the susceptibility to treatment and monitoring of treatment response (Figure 2) [109].

Initial evidence from in vitro models exists, indicating that SPIONs are not simply endocytosed by macrophages, but internalized depending on their interaction with proteoglycans expressed on the cellular membrane of macrophages, also referred to as glycocalyx. Specifically, Poller et al. demonstrated the binding of very small SPIONs to proteoglycans in the glycocalyx of monocytes and the occurrence of particle clusters reflecting their interaction prior to internalization. This finding highlights the importance of proteoglycans as components of the TME when developing new molecular imaging tools, because they potentially interfere with the imaging probes and affect their sensitivity and specificity [110].

In addition to capturing the immune landscape, there are theranostic applications of SPIONs as well, such as the intraarterial locoregional infusion of SPIO-labeled NK cells, which can be visualized with MRI and limit tumor progression [111]. SPIONs can also be incorporated into a complex with small interfering RNA, for targeted PD-L1-knockdown therapy combined with MRI diagnosis [112].

#### 2.4.2. Immunometabolic Crosstalk

Another MR application has recently been developed, which explores the concept of compound labeling commonly known from nuclear medicine tracers. Antibodies targeting surface receptors on antigen-presenting immune cells, such as human leukocyte antigens (HLA)-DR, were conjugated to gadolinium, and the conjugate was injected into liver tumor-bearing rabbits. The animals underwent 3T MRI, where select binding of the conjugate to peritumoral immune cells could be visualized as peritumoral ring enhancement in T1-weighted Dixon sequences. The imaging findings were validated by imaging mass cytometry on paraffin-embedded tumor tissue, which confirmed the gadolinium deposition in the peritumoral zone. Using the same approach combined with the previously explained pH-specific BIRDS MRSI, low tumor pH_e_ was correlated with an immunosuppressive microenvironment on imaging. Accordingly, increased tumor pH_e_ induced by TACE combined with sodium bicarbonate injection in vivo achieved better peri- and intratumoral immune cell infiltration, indicative of an immune permissive TME, that may potentially be more susceptible to immuno-oncologic therapies (Figure 2) [34].

## 3. Conclusions and Future Prospects

Altered cellular metabolism is a key feature of HCC that has a major impact on the tumor cells, tumor stroma, and local immune cells, and can be targeted by imaging and therapeutic approaches. Overall, there is an urgent clinical need for non-invasive characterization of the individual tumor and its TME, making the tumor metabolism and flux of metabolic substrates in the TME a promising target. The integration of molecular imaging of the tumor metabolism and its interaction with the various components of the TME in the clinical workup will ultimately help in applying and optimizing various targeted systemic, surgical or locoregional therapeutic approaches that have been already developed and will guide the development of new targeted therapies. A variety of functional imaging techniques exist, ranging from PET with traditional or new radiotracers to MR-based methods, and which complement anatomical imaging for the detection, diagnosis, staging, prediction, and monitoring of treatment responses in HCC. MR-based imaging modalities, which overcome some of the limitations of PET imaging, have been studied mostly in the preclinical setting (Table 1). However, the majority of approaches currently under investigation demonstrate limited sensitivity as well as low cost-efficiency, preventing their broad application in the management of HCC patients. Moreover, studies are absent that investigate the sensitivity and specificity of quantitative imaging biomarkers in the context of the histopathological and genomic tumor profile, despite the high number of identified driver genes and known inter- and intratumoral heterogeneity in HCC. Additionally, despite the targeted approach, many PET- and MR-based techniques alone do not sufficiently capture the complexity of the metabolic phenotype in HCC or its interaction with the stromal components and infiltrating immune cells in the TME, requiring combinations of several imaging modalities and targets to achieve higher diagnostic accuracy in a multiparametric approach. Therefore, future research endeavors in the molecular imaging of HCC should focus on the non-invasive monitoring of the crosstalk between the tumor cells and their TME mediated through tumor metabolism. In addition to improved tumor detection, such imaging tools may be able to provide a more accurate characterization of the individual tumor, and ultimately improve the understanding and facilitate the design of personalized treatment regimens for patients with liver cancer.

## Figures and Tables

**Table 1:** Overview of imaging techniques targeting the crosstalk between the tumor cells and their tumor microenvironment mediated through tumor metabolism and their level of evidence and clinical translation in HCC. BOLD, blood-oxygen-level-dependent MRI; CEST, chemical exchange saturation transfer; ECM, extracellular matrix; FDG, fluorodeoxyglucose; Gd-HLA-DR, gadolinium-conjugated antibodies against HLA-DR; GPC3, glypican-3; HCC, hepatocellular carcinoma; MRI, magnetic resonance imaging; MRSI, magnetic resonance spectroscopic imaging; PET, positron emission computed tomography; SPIONs, superparamagnetic iron oxide nanoparticles; TOLD, tissue oxygenation level-dependent MRI.

Superordinate Imaging Target		Imaging Technique	Imaging Target	Level of Evidence/Clinical Translation	References
I Imaging of metabolic substrates and flux	Glycolysis	^18^F-FDG-PET	Glucose	Clinical studies, experimental use (in HCC); Clinical resolution; FDG retention varies between different HCCs; Background signal in liver parenchyma; Limited spatial resolution (5 mm).	[17,18]
		1-^13^C- pyruvate MRSI	Pyruvate → Lactate/Alanine	In vivo studies in a rat model of HCC. Dynamic imaging; Clinical spatial resolution is lower; Higher dose of hyperpolarized substrates needed as compared to radionuclides, which may interfere with biologic pathways; Low spatial resolution.	[19,20]
	Lipid Metabolism	^11^C-choline PET	Choline	Clinical studies, experimental use; Application in a dual tracer approach.	[21,22]
		^11^C-acetate PET	Acetate	Clinical studies, experimental use; Application in a dual tracer approach.	[23,24]
	Amino Acid Metabolism	5-^13^C(1)-glutamine MRSI	Glutamine	In vivo study (rat model of HCC); Dynamic imaging; Low spatial resolution.	[25]
II Tumor microen- vironment	Extracellular pH	CEST	pH	Clinical studies, experimental use.	[26,27]
		BIRDS (MRSI)	pH	In vitro and in vivo studies in a rabbit liver tumor model; Low spatial resolution.	[5,28]
		acidic pH-triggered drug-release of sorafenib and superparamagnetic iron oxide nanocomposites	Low pH	In vivo studies in a mouse xenograft model of HCC. Theranostic approach.	[29]
		^18^F-FDG amine PET	pH	In vivo studies in a mouse xenograft model; Selectively degrades to ^18^F-FDG in an acidic environment, aims to reduce background signal, e.g., in the liver.	[30]
	Hypoxia	TOLD / BOLD MRI	Hypoxia	Clinical studies, experimental use.	[31]
	ECM	GPC3- labeled ^89^Zr PET	ECM/GPC3	In vivo mouse xenograft model.	[32]
III Inflammation	Immune evasion	SPIONs	Macrophages NK-cells	Clinical study, experimental use; High resolution.	
		CD8-minibodies PET	CD8 positive T cells	Clinical study, experimental use.	[33]
	Immuno-metabolic crosstalk	MRI with Gd-HLA-DR	Antigen-presenting cells	In vivo rabbit liver tumor model; High resolution.	[34]
